# Transparent 3-Layered Bacterial Nanocellulose as a Multicompartment and Biomimetic Scaffold for Co-Culturing Cells

**DOI:** 10.3390/jfb16060208

**Published:** 2025-06-03

**Authors:** Karla Pollyanna Vieira de Oliveira, Michael Yilma Yitayew, Ana Paula Almeida Bastos, Stefanie Cristine Nied Mandrik, Luismar Marques Porto, Maryam Tabrizian

**Affiliations:** 1 Department of Chemical Engineering and Food Engineering, Technology Center, Federal University of Santa Catarina (UFSC), Campus Reitor João David Ferreira Lima, Florianópolis 88040-900, SC, Brazil; 2 Faculty of Dental Medicine and Oral Health Sciences, McGill University, 2002 Avenue McGill College, Suite 500, Montreal, QC H2A 1G1, Canada; 3 BiomatX Lab, Department of Biomedical Engineering, Faculty of Medicine and Health Sciences, McGill University, 3775 University Street, Montreal, QC, H3A 2B6, Canada; 4 Embrapa Swine and Poultry, BR153, km 110, Tamanduá District, Concórdia 89715-899, SC, Brazil

**Keywords:** bacterial nanocellulose, 3D cell culture model, triple-cell co-culture

## Abstract

Three-dimensional (3D) cell culture models are widely used to provide a more physiologically relevant microenvironment in which to host and study desired cell types. These models vary in complexity and cost, ranging from simple and inexpensive to highly sophisticated and costly systems. In this study, we introduce a novel translucent multi-compartmentalized stacked multilayered nanocellulose scaffold and describe its fabrication, characterization, and potential application for co-culturing multiple cell types. The scaffold consists of bacterial nanocellulose (BNC) layers separated by interlayers of a lower density of nanocellulose fibers. Using this system, we co-cultured the MDA-MB-231 cell line with two tumor-associated cell types, namely BC-CAFs and M2 macrophages, to simulate the tumor microenvironment (TME). Cells remained viable and metabolically active for up to 15 days. Confocal microscopy showed no signs of cell invasion. However, BC-CAFs and MDA-MB-231 cells were frequently observed within the same layer. The expression of breast cancer-related genes was analyzed to assess the downstream functionality of the cells. We found that the E-cadherin expression was 20% lower in cancer cells co-cultured in the multi-compartmentalized scaffold than in those cultured in 2D plates. Since E-cadherin plays a critical role in preventing the initial dissociation of epithelial cells from the primary tumor mass and is often downregulated in the tumor microenvironment in vivo, this finding suggests that our scaffold more effectively recapitulates the complexity of a tumor microenvironment.

## 1. Introduction

Cells grow in a three-dimensional (3D) environment, surrounded by other cells and by the extracellular matrix (ECM), which is a complex and dynamic acellular network primarily composed of proteins and polysaccharides [1,2,3,4]. The ECM plays a vital role in cell development, differentiation, and tissue homeostasis, influencing gene expression and signaling pathways [2,5,6,7]. Its physical properties can change in response to disease, such as the increased stiffness observed in cancerous tissue [5,8].

To better mimic the characteristics of the ECM, cell culture models have become essential tools in the fields of cancer research, tissue engineering, and drug discovery. Knowing that the cells in two-dimensional (2D) environments [8,9,10,11] exhibit different growth patterns and motility compared to those in 3D systems [9,10], the current research focuses on the development of 3D cell models to more adequately mimic the complex and dynamic microenvironments of living tissues [7,10,11]. In contrast to the 3D model, cells in the 2D model grow in monolayers, have a higher proliferation rate and equal access to nutrients, typically adopt an elongated morphology, and tend to show greater sensitivity to drugs that are completely different from their native environment [10,11,12,13]. As a result, 3D models are increasingly recognized for their ability to mimic the tumor microenvironments (TMEs) and to investigate cancer progression, metastasis, and therapeutic resistance [14,15,16].

Several factors need to be considered when designing a 3D cell culture model. In addition to mechanical properties, porosity, pore interconnectivity and biocompatibility [17,18,19,20], the ability of the model to mimic the biological tissue microenvironment, and cell–cell interactions should also be considered.

Among the various materials used for 3D cell culture, hydrogels have emerged as promising scaffolds due to their ability to mimic key properties of the native ECM [21,22]. Their mechanical properties are similar to those of soft tissues [23], they are able to sequester and retain proteins within their structure, and they support cell adhesion. Among hydrogels, bacterial nanocellulose (BNC) is an ideal candidate for biomimetic scaffolds. BNC is a naturally derived, inert hydrogel composed of D-glucose chains secreted by Gram-negative bacteria of various genera such as *Komagataeibacter*. These bacteria produce long, non-aggregated fibrils up to 100 nm in diameter that are characterized by high purity and crystallinity [24,25,26,27].

The physical structure of BNC is similar to that of ECM and possesses some key properties relevant to tissue engineering, including high crystallinity (84–89%) [28], tensile strength (79–88GPa), and a water-holding capacity of 99% [24,29,30]. Moreover, the properties of BNC can be tailored when used as a composite scaffold to enhance cell adhesion and differentiation [31]. For instance, surface modifications such as the adsorption of (Ile-Lys-Val-Ala-Val) IKVAV peptide [32], collagen [33], or alginate [34] can significantly improve the cell adhesion.

BNC is a cost-effective polymer with high biocompatibility and non-toxicity [34,35,36]. It has been used as a scaffold for the culture of various cell lines, including HUVECs [37], SMCs [24], hASCs and iPSCs [38], chondrocytes [34], fibroblasts [39], neuroblastoma SH-SY5Y cells [33], and human skeletal muscle myoblasts [40], among many others.

Typically, BNC is produced by static bacterial culture and exhibits two distinct surfaces with different fiber densities. The air–liquid interface is more entangled and denser, while the liquid–contact interface is more porous [24,37]. This structural variation allows the fabrication of multilayered scaffolds where different cell types are cultured in separate compartments, allowing for the study of cell–cell interactions in a co-culture system [41,42]. Such co-culture systems are of particular interest for simulating the TME, where multiple cell types, including cancer cells, cancer-associated fibroblasts, and tumor-associated macrophages, interact to drive tumorigenesis and therapeutic resistance.

To date, research focusing on triple-cell co-cultures in BNC scaffolds, particularly in compartmentalized designs, remains largely unexplored, with few studies employing this technology to model complex, multi-cellular environments [43,44,45]. In this work, we introduce a transparent, compartmentalized, triple-layered BNC scaffold designed for the simultaneous co-culture of triple-negative breast cancer (TNBC) cells (MDA-MB-231), primary cancer-associated fibroblasts (CAFs) and tumor-associated macrophages (TAM, M2). By embedding each cell type in separate layers of the BNC scaffold, we have developed a multicompartment system that is expected to mimic the TME more accurately than traditional 2D or 3D models. Furthermore, we biomimetically characterized the material, assessed gene expression associated with TNBC metastasis, and demonstrated that the BNC scaffold supports long-term cell viability and metabolic activity.

## 2. Materials and Methods

### 2.1. Material and Chemical Origins

All chemical reagents utilized in this study were of analytical grade and procured from commercial suppliers unless specified differently. Mannitol, yeast extract, peptone, and agar were procured from Himedia^®^ (Kennett Square, PA, USA) for the formulation of Mannitol Agar medium. Sodium hydroxide (NaOH), ethanol (for dehydration processes), and paraformaldehyde were acquired from Sigma-Aldrich (Saint-Louis, MO, USA).

Culture media, including Dulbecco’s Modified Eagle Medium (DMEM, #11885-084), RPMI-1640 (#11875-093), and supplements such as fetal bovine serum (FBS, #FBS001), penicillin–streptomycin (#15140122), and β-mercaptoethanol (#M6250), were procured from Gibco (Thermo Fisher Scientific, Waltham, MA, USA) and Neuromics (Minneapolis, MN, USA) as indicated. CellTracker™ dyes (Invitrogen™/ThermoFisher Scientific, San Diego, CA, USA, #C34552, #C7025, #C2110), the Live/Dead Viability/Cytotoxicity Kit (Biotium, Fremont, CA, USA, #30002-T), and MTS assay reagents (Promega, Madison, WI, USA, #G358A) were utilized for cellular labeling and viability assessment. TRIzol™ reagent (ThermoFisher Scientific, #15596026) was employed for RNA extraction. All solutions were formulated with ultrapure water (Milli-Q^®^, Millipore/Sigma-Aldrich, San Francisco, CA, USA).

### 2.2. Multilayered BNC Fabrication

An aliquot of *K. hansenii* (ATCC 23769) was obtained from the Fundação André Tosello—Coleção de Culturas Tropical (Campinas, SP, Brazil) and expanded by culturing these bacteria in a Petri dish on Mannitol Agar medium (10 g/L mannitol, 2 g/L yeast extract, 1.2 g/L peptone, and 6 g/L agar) for 7 days under static conditions at 26 °C in BOD (Novatecnica, Piracicaba, SP, Brazil, model NT705).

**Figure 1:**
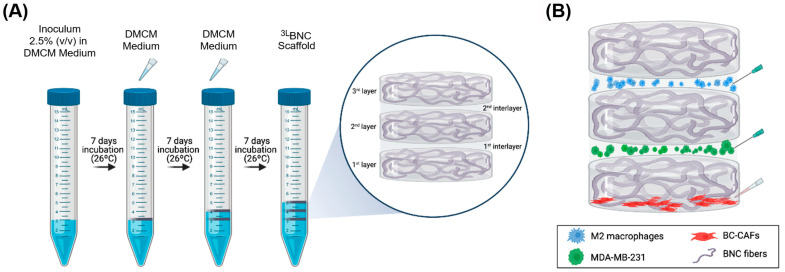
Schematic of ^3L^BNC scaffold fabrication and triple-cell co-culture. (**A**) Three-layer structure after 21 days of incubation in DMCM. (**B**) Cell injection/seeding protocol into each interfacial layer of ^3L^BNC scaffold for confocal microscopy analysis. A total of 9.4 × 10^4^ BC-CAFs were seeded under the first layer, then 2 × 10^6^ MDA-MB-231 and 9.3 × 10^4^ M2 macrophages were injected into the first and the second interlayer, respectively.

To fabricate the multilayered scaffolds, an inoculum with an optical density of 1–1.3 (Thermoplate, λ = 630 nm, Thermo Fisher Scientific) was prepared by diluting a few colonies of *K. hansenii* in Defined Minimal Culture Medium (DMCM) [46], followed by bacteria lysis using vortexing at maximum speed. This inoculum was then diluted to 2.5% (*v*/*v*) in DMCM. Three milliliters of this dilution was distributed in 15 mL conical polystyrene tubes and incubated under static conditions at 26 °C to form the first layer of the scaffold (Figure 1A). After 7 and 14 days of incubation, 750 µL of DMCM was carefully pipetted into the tubes to form the second and third layers, respectively. To eliminate bacteria, samples were incubated in 0.1 M sodium hydroxide (NaOH) at 50 °C for 24 h, followed by multiple rinses with distilled water until the pH reached 6.5. The samples were then sterilized at 121 °C for 20 min.

The above protocol was repeated to produce a sufficient number of samples for various analyses, with the desired layers of BNC (Table 1). For some analyses, the individual layers of the ^3L^BNC scaffold were separated, with the order indicated as 1 being the bottom layer and the 3 being the top layer.

### 2.3. Thickness and Transparency Measurement

The ^3L^BNC samples were carefully dried with absorbent paper towels before the thickness was measured using a digital micrometer (Mitutoyo 293-561-30, Mitutoyo Corporation, Kanagawa, Japan). After measuring the total scaffold thickness, the layers were separated individually with forceps, measured, and compared to the total scaffold. All measurements were made in triplicate using two independent batches (n = 6). The height of each interlayer was calculated using the following equation:

(1) interlayerthickness=3LBNCthickness−∑individual layerthickness ninterlayer

where ^3L^BNC_thickness_ is the thickness of the entire 3-layer BNC, ∑*individual layer_thickness_* refers to the sum of the thickness of each individual layer of ^3L^BNC, and n_interlayer_ is the number of interlayers in the samples.

To assess the transparency of the samples, the transmittance of the ^SL^BNC control, ^2L^BNC, and ^3L^BNC samples was measured using a spectrophotometer (λ = 550 nm), according to the method described by Saito et al. [47]. The average thickness of the samples was used for transparency conversion.

### 2.4. Pore Size Analysis

The ^3L^BNC samples were dehydrated using an increasing ethanol gradient (20–100%, 15 min each), followed by supercritical drying. After drying, the samples were immersed in liquid nitrogen (N_2_), cross-sectioned, and prepared for Scanning Electron Microscopy (SEM). SEM analysis was conducted on three samples from two different batches of BNC (n = 3) using a JEOL JSM-6390LV microscope (JEOL, Akishima, Tokyo, Japan), with images captured at an acceleration voltage of 10 kV. The pore size of the scaffold was determined using ImageJ software (version 1.52) from SEM images at 5000× magnification, using the ‘analyze particles’ function of the software. The Feret diameter was then compared among samples.

### 2.5. Nutrient Transport

To evaluate whether ^SL^BNC and ^2L^BNC samples allowed glucose transport, a protocol adapted from Papenburg et al. [48] was used. Briefly, two polystyrene flasks were connected by a 5 mm hole and sealed with the samples. The upper flask contained a 1 g/L glucose solution, while the lower flask was filled with glucose-free liquid. The glucose concentration in both containers was determined using an enzymatic PGO assay (Sigma-Aldrich, #P7119-10CAP) with a spectrophotometer (SpectraMax i3 Platform, Molecular Devices, San Jose, CA, USA; λ = 450 nm). Calculations were performed as described by the authors. To simulate cell culture conditions, 20 mL of supplemented DMEM (Gibco, #11885092) was added to the donor flask, and an equal volume of 1× PBS was added to the receptor tube. Glucose solution and deionized water were used as controls in the donor and receptor flasks, respectively. Samples of 0.5 mL were taken from both containers at t = 0 (baseline), and at 15 min, 30 min, 1 h, 2 h, 4 h, 8 h, and 24 h intervals, unless there was no remaining volume in the donor flask.

### 2.6. Rheological Analysis

The ^2L^BNC and ^3L^BNC samples were characterized using uniaxial compression tests and shear stress analysis via a torsional rheometer (Discovery HR-2, TA Instruments, New Castle, DE, USA) equipped with a 20 mm circular plate positioned on a Peltier stage set at 37 °C. Compression data were obtained by applying 90% compressive strain at a rate of 10 µm/s. Young’s modulus (E) was calculated from the strain–stress curves in the 5–10% strain range.

Amplitude sweep tests were performed at an angular frequency of 10 rad/s over a strain range of 0.01% to 500%, at a constant temperature of 37 °C, with 10 data points collected per decade. The storage modulus (G’) of the ^2L^BNC and ^3L^BNC samples was obtained from the linear portion of the oscillatory strain/module curve. All tests were performed in triplicate and analyzed using TRIOS software (version 4.5.0.42498).

### 2.7. Standardization of Cell Culture Protocol

The cell culture was standardized in ^2L^BNC and ^3L^BNC prior to triple co-culture (Table 1). EOMA (mouse hemangioendothelioma endothelial cell line, ATCC #CRL-2586), EA.hy926 (human umbilical vein cell line, BCRJ #0345), and MDA-MB-231 (ATCC #HTB-26) cells were cultured in DMEM (Gibco, #11885-084) supplemented with 10% fetal bovine serum (FBS, Neuromics, #FBS001) and 1% penicillin–streptomycin (Pen/Strep, Gibco, #15140122). BC-CAFs (Neuromics, #CAF116) were maintained in the manufacturer’s recommended medium (MSC-GRO^®^, Neuromics, #PC00B1).

The THP-1 cell line was kindly provided by Dr. Cerruti’s lab (ATCC, TIB-202) and differentiated into M0 macrophages [49], and subsequently polarized into M2 cells [50]. Both monocytes and macrophages were cultured in RPMI-1640 (Gibco, #11875-093) supplemented with 10% FBS, 1% Pen/Strep, and 50 µM β-mercaptoethanol (Sigma-Aldrich, #M6250), followed by 0.22 µm filtration (Sigma, #S2GVU05RE).

All cultures were maintained in humidified incubators at 37 °C with 5% CO_2_, and the corresponding media were renewed every 2–3 days. Cells were plated at greater than 80% confluency, after which the MDA-MB-231 conditioned medium (CM) was prepared by 0.22 µm media filtration.

### 2.8. Cell Viability

The live/dead assay for EOMA cells cultured on the ^2L^BNC and ^3L^BNC scaffolds was performed according to the manufacturer’s instructions (Biotium, #30002-T). Images were captured using a Nikon Eclipse TE2000-U (Nikon, Melville, NY, USA) inverted microscope with NIS Element D 4.11.00 software. To assess the cell metabolic activity, the MTS assay (Promega, #G358A) was performed at 3, 5, 7, 10, and 15 days after injection of MDA-MB-231 cells into ^2L^BNC samples (Table 1). Data from cells grown in 24-well plates (8 × 10^4^ cells/well) at the same time points were used as a reference. Three biological and three technical replicates were used for comparison. Absorbance was recorded at λ = 490 nm using a spectrophotometer. Cell growth was also visualized using a TE2000-U microscope.

### 2.9. Triple Co-Culturing into ^3L^BNC Scaffolds

First, 1 × 10^6^ EA.hy926 cells were injected into the second interlayer of the ^3L^BNC scaffolds to confirm that the cells could be maintained within the compartments of the scaffold. Cells were maintained in standard cell culture inserts for 15 days under the same conditions as above. On days 1, 5, 10, and 15, the scaffolds were rinsed twice with 1 mL of 1X PBS, fixed with 4% paraformaldehyde for 1 h, and stained with DAPI at a dilution of 1:2000 for 30 min. Cells within the scaffold were then imaged using an inverted phase microscope (Carl Zeiss, Jena, Germany, Axio Vert. A1) and analyzed using FIJI (version 2.14.0/1.54f). For the triple co-culture, a conditioned medium (1:1) was used to prime BC-CAFs for at least two days before their seeding at the bottom of ^3L^BNC samples. This medium was maintained until MDA-MB-231 cells were injected into the first interlayer (Figure 1B). MSC-GRO^®^/DMEM (1:1) was used to replace the CM immediately after the injection. When M2 macrophages were injected into the second interlayer (Figure 1B), the system was maintained in MSC-GRO^®^/DMEM/RPMI-1640 (1:1:1) medium.

Confocal microscopy and gene expression analysis were performed at 1, 5, 10, and 15 days after cell seeding/injection. Samples were maintained in non-treated 24-well culture plates throughout the analysis period and were transferred to new wells on the day of media replacement. Injections were performed using a 25G 1½ inch needle (BD, Franklin Lakes, NJ, USA, #305127).

### 2.10. Confocal Microscopy

Red-stained (Invitrogen, #C34552) BC-CAFs were seeded at the bottom of the scaffold and incubated for 2 days prior to injecting of green-stained MDA-MB-231 cells (Invitrogen, #C7025). After an additional 2 days (4 days total since BC-CAF seeding), blue-stained M2 macrophages (Invitrogen, #C2110) were injected into the ^3L^BNC samples. All cell trackers were used at a concentration of 25 µM. At the appropriate time point, samples were removed, rinsed twice with 1 mL of 1× PBS, and fixed with 4% paraformaldehyde for 1 h. Samples were then washed three times in 1× PBS (5 min each), mounted with Aqua-Poly/Mount (Polysciences, Warrington, PA, USA, #18606-20), and stored at 4 °C until imaging using the ABIF Opera Phenix High Content Screening system with a 5× objective and a 20× water immersion objective, at 30 µm and 15 µm intervals, respectively.

### 2.11. RNA Quantification by qPCR

MDA-MB-231 and M2 cell cultures were injected into the scaffolds at intervals of 3 and 7 days, respectively, after BC-CAF seeding. RNA was extracted using TRIzol™ (ThermoFisher Scientific, #15596026) according to the manufacturer’s instructions. RNA concentration and purity were assessed using a Nanodrop spectrophotometer (ThermoFisher Scientific). Expression of breast cancer-associated genes was normalized to *GAPDH* and evaluated by RT-qPCR (Promega, #A6010) using three replicates per time point. The primers listed in this study (Table 2) were used at a concentration of 100 µM.

### 2.12. Statistical Analysis

Two-way analysis of variance (ANOVA) with Tukey’s post hoc test was used to compare multiple groups for Feret diameter. Two-way ANOVA with Šidák correction was used to compare gene expression between groups. Data are presented as the mean ± standard deviation of results from three independent experiments unless otherwise specified. *p* < 0.05 was considered statistically significant (* 0.01 < *p* < 0.05, ** 0.001 < *p* < 0.01, *** *p* < 0.001, and **** *p* < 0.0001). The Pearson correlation test was used to correlate individual layers. All analyses were performed using GraphPad (version 9.1.0.221).

## 3. Results

### 3.1. Physicochemical Properties of the ^3L^BNC Scaffold

**Figure 2:**
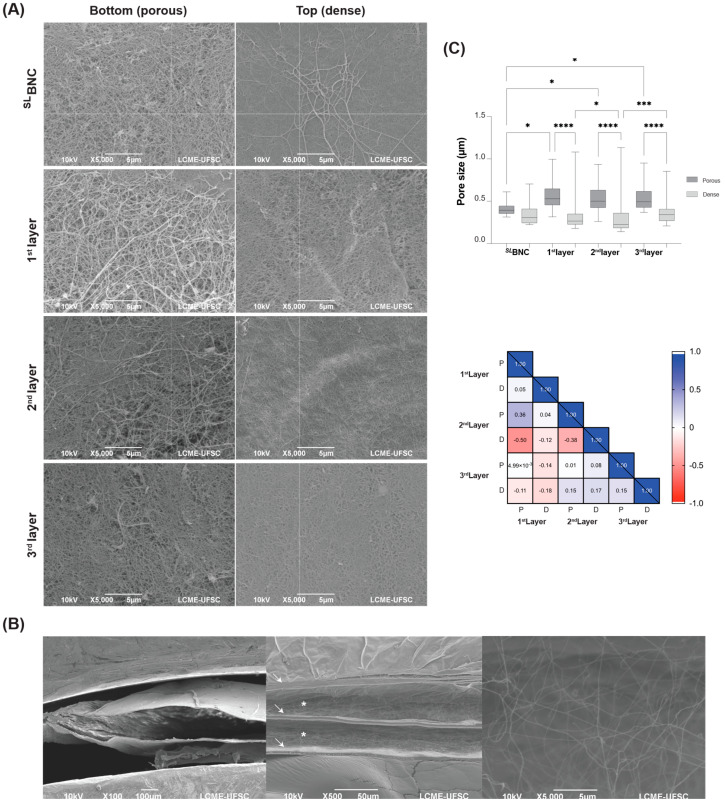
Microstructure and pore size of ^3L^BNC scaffolds. (**A**) SEM micrographs of individual layers of ^3L^BNC compared to the ^SL^BNC control showing a more porous structure for the bottom layer (5000×). (**B**) Cross-section of ^3L^BNC showing layers (arrows) and interlayers (*) at 100×, 500×, and 5000× magnification. (**C**) Feret diameter of the ^3L^BNC-derived individual layers compared to ^SL^BNC and Pearson’s correlation matrix of individual layers’ surfaces: P: porous; D: dense. *n* = 1 for ^SL^BNC, and *n* = 3 for layers. * 0.01 < *p* < 0.05, *** *p* < 0.001, and **** *p* < 0.0001.

The ^3L^BNC samples had an average thickness of 7.0385 ± 0.6146 mm. The thicknesses of the individual layers were 1.975 ± 0.298 mm, 2.030 ± 0.273 mm, and 1.979 ± 0.315 mm for the first, second, and third layers, respectively. These values are comparable to the ^SL^BNC control, which had a thickness of 2.312 ± 0.471 mm. SEM micrographs showed different nanofiber densities, resulting in porous (bottom) and dense (top) surfaces in each individual layer, similar to the ^SL^BNC control (Figure 2A). In addition, differences in pore sizes were observed between the surfaces of each individual layer and across different layers. However, these differences were not significantly correlated, suggesting that the fibers on both surfaces are randomly arranged on both surfaces (Figure 2B and Table S1).

Regarding the interlayers, they showed a height of 0.4157 ± 0.0396 mm and featured larger interconnected pores with an average size of 2.386 ± 0.981 µm (Figure 2C). The transparency of the ^SL^BNC control was twice that of the ^2L^BNC and four times that of the ^3L^BNC scaffolds, 31.3 ± 4.2% vs. 14.2 ± 1.3%, respectively (Figure S1).

The rheological properties of ^2L^BNC and ^3L^BNC are reported in Table 3 and Figure S2. The ^3L^BNC exhibited a 75% higher stiffness (E) and a 36.5% higher storage modulus (G) compared to ^2L^BNC (*p* < 0.05), indicating that increasing the number of layers directly affects the viscoelastic properties of the scaffold.

### 3.2. Cellular Viability and Proliferation of Cells Cultured in Layered BNC Scaffolds

**Figure 3:**
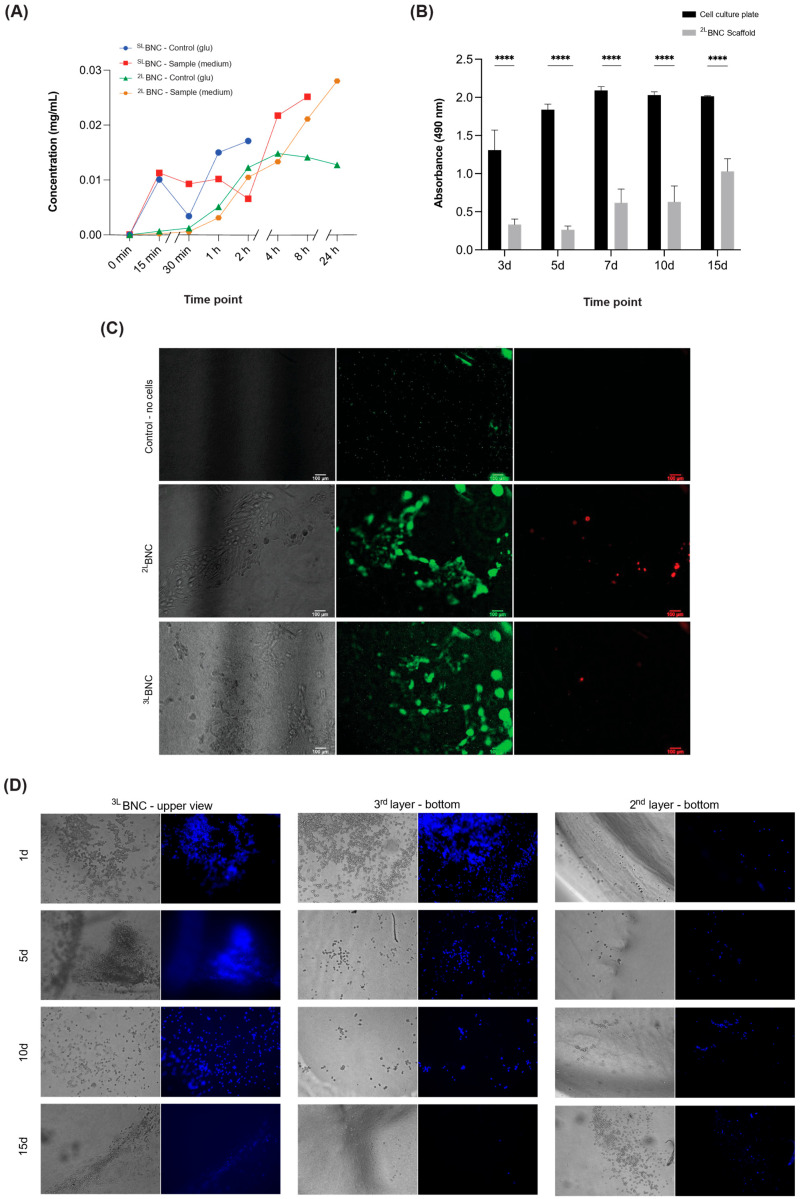
Evaluation of cell culturing into layered scaffolds. (**A**) Diffusion assay in ^2L^BNC compared to ^SL^BNC. DMEM tested and compared to glucose solution in both scaffolds. (**B**) Cell proliferation (MTS) assay with MDA-MB-231 in ^2L^BNC samples and in 2D cell culture plate (2 × 10^6^ cells/scaffold 3D culture and 8 × 10^4^ cells/well 2D culture). One-way ANOVA (*p* < 0.05) with n = 9. (**C**) Live/dead assay with EOMA cells after one-week cell culture in the ^2L^BNC and ^3L^BNC scaffolds (live cells in green, dead cells in red, merged images in last column, scale bars: 100 µm). (**D**) DAPI assay of EA.hy926 cultured in second interlayer of ^3L^BNC scaffolds for up to 15 days. Samples remained inside inserts to avoid lateral cell migration; from left to right: top view of entire scaffold, bottom view of third and second layers. **** *p* < 0.0001.

It was assessed that both glucose solution and DMEM-supplemented medium can flow through the BNC scaffolds for up to 24 h (Figure 3A). EOMA cells cultured on ^2L^BNC or ^3L^BNC scaffolds were viable for up to one week (Figure 3C and Figure S3).

The MDA-MB-231 cells remained metabolically active for up to 15 days, as revealed by the MTS assay (Figure 3B). No differences in metabolic activities of cells were noticed after cell seeding/injection between days 3 and 5 or between days 7 and 10 of culture. Furthermore, for this reason, further analyses were performed after 1-, 5-, 10-, and 15-day seeding/injection of cells.

EA.hy926 cells injected in the second interlayer of the ^3L^BNC and imaged at the designated time points consistently showed the presence of these cells at the injected interfacial layer for the entire duration of analysis (Figure 3D and Figure S4). This was confirmed after separating the third and second layers and subsequently imaging their bottom surfaces. (*p* < 0.05).

### 3.3. Triple-Cell Co-Culture in ^3L^BNC Scaffolds

**Figure 4:**
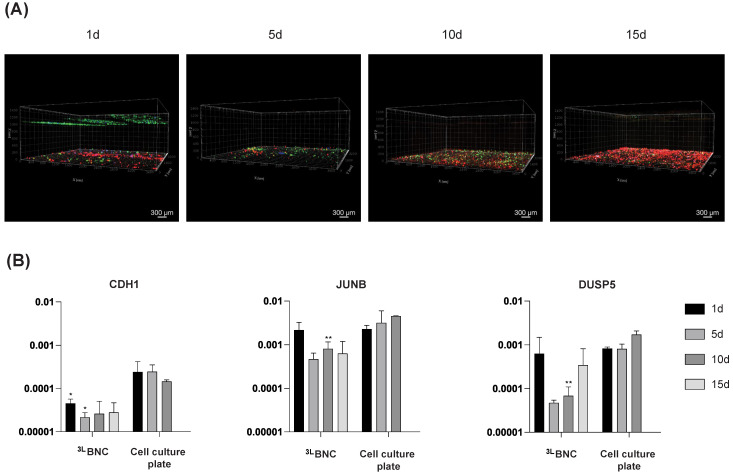
Triple-cell co-culture in ^3L^BNC scaffold. (**A**) Three-dimensional confocal microscopy of cells seeded/injected into ^3L^BNC at different time points: MDA-MB-231 in green, BC-CAFs in red, M2 macrophages in blue. (**B**) CDH1, JUNB, and DUSP5 gene expression relative to GAPDH. The 15-day time point plate-grown cells was not tested. Asterisks refer to statistical significance of cells cultured in the ^3L^BNC versus cells cultured on the plates (* 0.01 < *p* < 0.05, ** 0.001 < *p* < 0.01).

The cell migration assay was performed with the triple-cell co-culture of BC-CAFs, MDA-MB-231 cells, and M2 macrophages in different compartments of ^3L^BNC samples. Confocal microscopy on day 1 showed that BC-CAFs and MDA-MB-231 cells remained at their respective locations (Figure 4A). In contrast, blue-stained M2 macrophages injected into the second interlayer migrated rapidly toward other cells, as indicated by the colocalization of blue, red, and green colors in the confocal images. Interestingly, at day 5, MDA-MB-231 cells migrated to the BC-CAF injected site in the bottom layer of the ^3L^BNC. However, M2 macrophages were not easily distinguishable. To confirm the presence of M2 in the same layer, the confocal images were filtered for the blue color corresponding to macrophage staining. Except for day 5, all images for other time points exhibited blue dots consistent with M2 macrophage presence (Figure S5).

Breast cancer gene expression was measured at each time point, and relative expression levels were normalized to *GAPDH* (Figure 4B and Table S2). *CDH1* (E-cadherin) showed the lowest expression among all genes in the ^3L^BNC samples. *JUNB* exhibited similar expression levels between scaffolds and plates on day 1, but its expression decreased at later time points, although it remained the most highly expressed gene overall. *DUSP5* expression declined by day 5, followed by a gradual increase at later time points.

## 4. Discussion

We fabricated a non-functionalized, triple-layered BNC (^3L^BNC) scaffold that enables the compartmentalized culture of cells to study intercellular interactions and signaling between primary cancer-associated fibroblasts (CAFs), the triple-negative breast cancer cell line MDA-MB-231, and M2 macrophages, each seeded or injected into separate layers. CAFs are a heterogeneous population of activated fibroblasts [51] that promote tumor cells [52,53], enhance angiogenesis [53], confer drug resistance [54], and recruit and polarize monocytes into pro-tumor M2 macrophages [55]. Macrophages, in turn, participate in almost all stages of tumor progression, and their high infiltration in breast cancer is associated with poorer prognosis [56]. The synergistic interactions and crosstalk between CAFs and M2 macrophages have been highlighted in several recent reviews [57,58].

The translucency of the ^3L^BNC scaffold makes it compatible with a variety of imaging modalities, allowing us to visualize the cell morphology throughout the scaffold. Using bright-field microscopy, we observed that breast cancer cells exhibited a range of shapes, from round to stellate, over the course of the observation period, consistent with the typical morphology for these cells in soft hydrogels [59]. Interestingly, distinct morphologies of the same cell line were observed when analyzed by fluorescence confocal microscopy. This observation aligns with previous reports indicating that the MDA-MB-231 cells adopt a spherical or spindle-shaped morphology depending on the scaffold’s composition and stiffness [60], and that larger rounded cells are associated with higher metastatic potential [61].

Cell morphology depended on the BNC surface to which cells were exposed [24,37] and seeding density [37]. Human umbilical vein endothelial cells and smooth muscle cells exhibited more elongated shapes when cultured on an entangled BNC surface compared to the porous surface. The effect of cell number was evident in the stellate and spindle-shaped morphologies after 1 day after seeding, likely due to the lower number of BC-CAFs used compared to the MDA-MB-231 cells. Nevertheless, the observed morphologies were consistent with previously reported patterns [62], even when cells were cultured on the underside of the BNC, which features larger pores. These findings suggest that the ^3L^BNC scaffold supports diverse cell morphologies across its surface and that different cell types respond uniquely to its topography. Furthermore, the morphological features of MDA-MB-231 cells in the ^3L^BNC scaffold are indicative of high metastatic potential.

Scaffold pore size plays a pivotal role in cell morphology [63], cell growth [64], nutrient supply [65], ECM secretion [65], cell invasion [66], and gene expression [63]. We observed significant differences in pore size between layers and interlayers. While interlayer compartments featured pores in the micrometer range, the pores within each layer were in the nanometer range. The lack of correlation between pore sizes across the layers supports the structural independence of each layer. Consequently, the scaffold effectively accommodates and compartmentalizes distinct cell populations. This was exemplified in the culture of EA.hy926 cells, which largely remained localized at the injection site, with minimal migration into other regions of the scaffold.

Similarly, the MDA-MB-231 cell line remained compartmentalized on day 1. However, over time, both the MDA-MB-231 cells and M2 macrophages migrated toward the first layer containing BC-CAFs. This behavior can be explained by several key factors.

First, the term “invasion” traditionally refers to the destructive movement of cells through a 3D barrier [67]. However, since human cells lack cellulase, the cell movement observed in our scaffold is more accurately described as “3D migration”, a non-destructive, non-proteolytic process in which cells traverse 3D tissues without degrading the matrix, as described by Kramer et al. [67].

Second, the MDA-MB-231 cells are highly motile and invasive, particularly in the presence of CAF conditioned medium, in both two- and three-dimensional environments [68]. Additionally, cell migration is further supported by the presence of different CAF subtypes, which are commonly found in primary and metastatic tumor stroma and possess varying capacities to migrate and to create a pro-tumorigenic microenvironment that facilitates cancer cell movement [69,70].

Third, M2 macrophages typically display lower adhesion and higher motility compared to classically activated macrophages [71] and they are often found near CAFs [55]. It is important to consider the source of macrophages, as their phenotype can vary significantly. The THP-1-derived macrophages used in this study may have different characteristics compared to bone marrow-derived macrophages [72]. Thus, the observed cell motility within the ^3L^BNC scaffold may be attributed to cell detachment and migration along signaling gradients established by the scaffold’s multilayered architecture.

Fourth, mechanical properties are also key regulators of cell migration. Cells modulate their migration speed according to substrate stiffness [73], which varies across healthy tissues [74] and is frequently altered in pathological conditions [75]. In our study, the stiffness of our scaffold increased when comparing ^2L^BNC and ^3L^BNC. This increase mirrors the mechanical characteristics of tumor-associated stroma and premalignant breast tissue [76], suggesting that the ^3L^BNC scaffold provides a physiologically relevant platform for studying breast tumor behavior.

Finally, pore size and interconnectivity are critical factors in cell migration. Unlike the cellular ingrowth observed for HUVEC cells in BNC hydrogels [37], confocal images of our ^3L^BNC system did not reveal the presence of cells within the scaffold layers, likely due to their smaller pore size compared to the interlayer compartments. Nevertheless, our findings indicate that the pore size of the ^3L^BNC scaffold is adequate to support nutrient diffusion and maintain cell viability within the interlayers.

Both pore size and mechanical properties influence gene expression [63,77], primarily through cell mechanosensing. To assess the impact of the ^3L^BNC on gene expression, we evaluated the levels of E-cadherin, *JUNB*, and *DUSP5*, three genes associated with breast cancer. E-cadherin (*CDH1*) encodes a cell–cell adhesion protein of the same name, and its downregulation is linked to increased invasiveness and metastasis [78,79,80,81]. MDA-MB-231 cells, a highly proliferative phenotype [82] and an E-cadherin-negative triple-negative breast cancer cell line, typically exhibit low *CDH1* expression. Consistent with this phenotype, we observed lower *CDH1* expression in cells cultured within ^3L^BNC compared to the plate-cultured controls. This suggests that the scaffold effectively recapitulates the complexity of the TME, aligning with the enhanced migratory behavior of MDA-MB-231 cells observed in our system.

The enhanced metastatic behavior of MDA-MB-231 cells cultured in ^3L^BNC is further supported by our findings on *JUNB*, a gene encoding a key AP-1 family transcription factor induced by TGF-ß [83,84]. *JUNB* plays a dual role in regulating cell proliferation, acting as both a promoter and an inhibitor, depending on cellular context [85]. It is also essential for breast cancer invasion, progression, and metastasis [83], and is known to be highly expressed in circulating tumor cells [86,87]. Transcriptomic analyses have previously confirmed elevated *JUNB* mRNA levels in the MDA-MB-231 cell line [86,87]. In our study, *JUNB* was the most highly expressed gene of the three genes, with expression levels approximately 16% and 10% higher than *CDH1* and *DUSP5*, respectively. However, *JUNB* expression was lower in cells cultured in the ^3L^BNC scaffold compared to standard well plates, suggesting that the scaffold recapitulates aspects of the tumor microenvironment that influence the fine-tuned regulation of gene expression.

Finally, we evaluated *DUSP5* expression due to its reported association with paclitaxel (PTX) resistance in basal-like breast cancer [88]. Dual-specificity phosphatases (*DUSPs*) comprise a family of 25 proteins that dephosphorylate threonine/serine residues of MAPK components and can function as either tumor suppressors or activators, depending on ERK signaling context and the specific *DUSP* family member [89]. In colorectal cancer, low *DUSP5* expression has been linked to poor prognosis. Moreover, a positive correlation between *DUSP5* and *CDH1* expression suggests a role for *DUSP5* in regulating epithelial–mesenchymal transition, a key process in metastasis [89]. In our study, *DUSP5* exhibited intermediate expression relative to *JUNB* and *CDH1*, and its expression was lower in cells cultured in the ^3L^BNC scaffold compared to conventional plate culture, further supporting the scaffold’s ability to replicate features of the in vivo tumor microenvironment.

It is important to note that the RNA yield extracted from cells cultured in BNC scaffolds was approximately three times lower than that obtained from cells cultured in standard well plates. This reduction is attributable to the hydrogel nature of BNC, which has a water-holding capacity of up to 99% [24,90]. In our study, the ^SL^BNC and ^2L^BNC scaffolds exhibited swelling degrees of approximately 86% and 200%, respectively. This high swelling capacity hinders the complete release of hydrophilic molecules, such as RNA, thereby complicating downstream molecular analyses. As a result, gene expression studies involving BNC scaffolds are often omitted in the literature.

## 5. Conclusions

This work lays the foundation for using triple-layered bacterial nanocellulose scaffolds as customizable, multifunctional platforms for modeling complex tumor microenvironments. We demonstrated that this translucent scaffold exhibits mechanical properties comparable to those of tumor-associated stroma and supports the compartmentalized co-culture of breast cancer cells, M2 macrophages, and cancer-associated fibroblasts. This design enabled spatially controlled co-culture and dynamic analysis of cell migration, offering a more physiologically relevant 3D model that overcomes key limitations of conventional transwell-based migration assays.

Differential expression of key metastasis-associated genes (*CDH1*, *JUNB*, and *DUSP5*) confirmed the scaffold’s ability to recapitulate important molecular features of the tumor microenvironment. Additionally, the scaffold’s optical transparency allowed for morphological assessment and real-time visualization of cell–cell interactions, typically not possible with standard in vitro systems.

The potential for chemical functionalization and structural customization further expands the scaffold’s utility in drug discovery and therapeutic development. While the aim here was to develop an in vitro model for mimicking the tumor microenvironment, its translucid properties also open avenues for investigating photosensitive treatments, such as cancer photothermal and photodynamic therapies. It is worth noting that bacterial nanocellulose is a biocompatible and scalable material already utilized in FDA-approved biomedical devices, such as wound dressings. This precedent may facilitate future regulatory translation, particularly if the scaffold is adapted for use in diagnostics, in therapeutic testing platforms, or as a 3D construct for in vivo applications.

## Figures and Tables

**Table 1:** Description of BNC stacked multilayered scaffolds and analysis for each.

	Analysis	^SL^BNC	^2L^BNC	^3L^BNC	Individual Layers	Type of Cell: Number
		Single-Layer (Control) (7 Days Incubation)	Double-Layer (14 Days Incubation)	Triple-Layer (21 Days Incubation)		When Applicable
Physical Characterization	Thickness	√	n/a	√	√	n/a
	Transparency	√	√	√	n/a	n/a
	Pore size	√	n/a	√	√	n/a
	Nutrient transport	√	√	n/a	n/a	n/a
	Rheology	n/a	√	√	n/a	n/a
Biological Characterization	Cell viability	n/a	√	√	n/a	EOMA: 10^4^ and 10^5^
	Cell metabolic activity	n/a	√	n/a	n/a	MDA-MB-231: 2 × 10^6^
	Cell migration	n/a	n/a	√	n/a	EA.hy926: 10^6^
	Confocal microscopy	n/a	n/a	√	n/a	BC-CAFs: 9 × 10^4^ MDA-MB-231: 2 × 10^6^ M2: 4 × 10^5^
	Gene expression	n/a	n/a	√	n/a	BC-CAFs: 9 × 10^4^ MDA-MB-231: 2 × 10^6^ M2: 4 × 10^4^

√: measurements and analysis performed; n/a: not applicable.

**Table 2:** List of primers and genes of interest.

NCBI ID	Gene	Forward/ Reverse	Sequence (5′ → 3′)	Start	End
NM_002046.7	*GAPDH*	F	CACCCACTCCTCCACCTTTG	943	963
		R	CCACCACCCTGTTGCTGTAG	1052	1032
NM_002229.3	*JUNB*	F	TTCAAGGAGGAACCGCAGAC	1001	1021
		R	TGAGCGTCTTCACCTTGTCC	1196	1176
NM_004419.4	*DUSP5*	F	CCAACTTTGGCTTCATGGGC	1120	1140
		R	GCTCAGTGTCTGCAAATGGC	1253	1233
Z13009.1	CDH1 *	F	GGTCTCTCTCACCACCTCCA	1483	1503
		R	GGATGTGATTTCCTGGCCCA	1615	1595

* Also known as E-cadherin.

**Table 3:** Scaffolds’ rheological properties.

	1st Layer	2nd Layer	3rd Layer	^2L^BNC	^3L^BNC
Young’s Module—E (Pa)	569.67 ± 515.39	869.34 ± 366.51	927.44 ± 102.29	183.61 ± 85.22	723.68 ± 306.53
Storage Modulus—G’ (Pa)	712.36 ± 0.00 ^a^	1756.44 ± 0.00 ^a^	382.66 ± 0.00 ^a^	2067.95 ± 237.98	3257.93 ± 450.07
Loss Modulus—G” (Pa)	125.74 ± 0.00 ^a^	219.17 ± 0.00 ^a^	53.64 ± 0.00 ^a^	309.21 ± 6.93	540.28 ± 66.09

^a^ Tests performed in only one sample. Different letters indicate significant differences.

## Data Availability

The original contributions presented in this study are included in the article and Supplementary Materials. Further inquiries can be directed to the corresponding author.

## Supplementary Materials

The following supporting information can be downloaded at: https://www.mdpi.com/article/10.3390/jfb16060208/s1, Figure S1: Image of ^SL^BNC control and ^3L^BNC scaffolds acquired by a stereoscope; Figure S2: Graphs presenting rheology properties of the BNC scaffolds form which the average Young’s modulus-E, Storage modulus–G’. Loss modulus-G” are calculated. *n* = 3 for each sample, except for Storage and Loss modulus of individual layers; Figure S3: Live/Dead assay of one-week EOMA cells culture into the ^2L^BNC (A) and into ^3L^BNC (B) scaffolds at two different densities (1 × 10^4^ and 1 × 10^5^ cells); Figure S4: EA.hy926 cultured in the ^3L^BNC Scaffolds; Figure S5: Images of M2 macrophages alone (first column) in comparison with their presence with all other cells simultaneously (second column) at different time-points: MDA-MB-231 in green, BC-CAFs in red and M2 macrophages in blue. Magnification 20×; Table S1: Feret diameter (µm) of porous and dense surfaces of each layer of ^3L^BNC scaffolds and ^SL^BNC control; Table S2: Nanodrop results from RNA extraction by Trizol of cells grown in 3LBNC Scaffold and cell culture plates. NC: negative control–no cells.
