# Supplementary material for: Transparent 3-Layered Bacterial Nanocellulose as a Multicompartment and Biomimetic Scaffold for Co-Culturing Cells
Source: J Funct Biomater. 2025 Jun 3;16(6):208. doi: 10.3390/jfb16060208 (PMC12194138; doi:10.3390/jfb16060208)
Supplement: Supplementary file 1 [file jfb-16-00208-s001.zip › jfb-3645772-supplementary.pdf]

Supplementary Information

Transparent 3-Layered Bacterial Nanocellulose as a Multicompartment and Biomimetic Scaffold for Co-Culturing Cells

Karla Pollyanna Vieira de Oliveira [1,2], Michael Yilma Yitayew [3], Ana Paula Almeida Bastos [4], Stefanie Cristine Nied Mandrik [1], Luismar Marques Porto [1] and Maryam Tabrizian [2,3,*]

[1] Department of Chemical Engineering and Food Engineering, Technology Center, Federal University of Santa Catarina (UFSC), Campus Reitor João David Ferreira Lima, Florianópolis 88040-900, SC, Brazil
[2] Faculty of Dental Medicine and Oral Health Sciences, McGill University, 2002 Avenue McGill College, Suite 500, Montreal, QC H2A 1G1, Canada
[3] Department of Biomedical Engineering, 740 Dr. Penfield, Montreal, QC H3A 0G1, Canada
[4] Embrapa Swine and Poultry, BR153, km 110, Tamanduá District, Concórdia 89715-899, SC, Brazil
* Correspondence: maryam.tabrizian@mcgill.ca

Table S1: Feret diameter (µm) of porous and dense surfaces of each layer of ^{3L}BNC scaffolds and ^{SL}BNC control

	**^{SL}BNC**		**1st layer**		**2nd layer**		**3rd layer**	
	**Porous**	**Dense**	**Porous**	**Dense**	**Porous**	**Dense**	**Porous**	**Dense**
**Number of values**	19	13	75	70	39	82	37	45
**Minimum**	0.3130	0.2236	0.3162	0.1789	0.2608	0.1414	0.3677	0.2088
**25% Percentile**	0.3544	0.2429	0.4561	0.2269	0.4238	0.1844	0.4265	0.2694
**Median**	0.3929	0.3053	0.5280	0.2668	0.5016	0.2258	0.4940	0.3418
**75% Percentile**	0.4441	0.4110	0.6478	0.3506	0.6315	0.3605	0.6187	0.4064
**Maximum**	0.6122	0.7034	0.9964	1.082	0.9364	1.133	0.9508	0.8528
**Range**	0.2992	0.4798	0.6802	0.9028	0.6756	0.9915	0.5831	0.6440
**Mean**	0.4084	0.3465	0.5568	0.3274	0.5416	0.2809	0.5371	0.3588
**Std. Deviation**	0.07974	0.1345	0.1439	0.1688	0.1714	0.1450	0.1495	0.1169
**Std. Error of Mean**	0.01829	0.03729	0.01661	0.02018	0.02744	0.01601	0.02458	0.01743
**Lower 95% CI of mean**	0.3700	0.2653	0.5237	0.2871	0.4860	0.2490	0.4873	0.3237
**Upper 95% CI of mean**	0.4468	0.4278	0.5899	0.3676	0.5971	0.3127	0.5870	0.3940

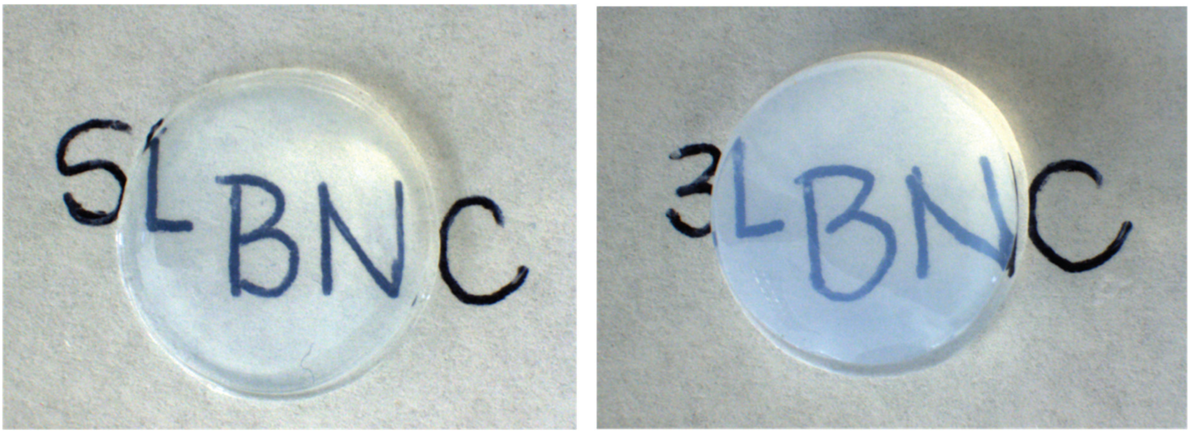

Figure S1: Image of ^{SL}BNC control and ^{3L}BNC scaffolds acquired by a stereoscope.

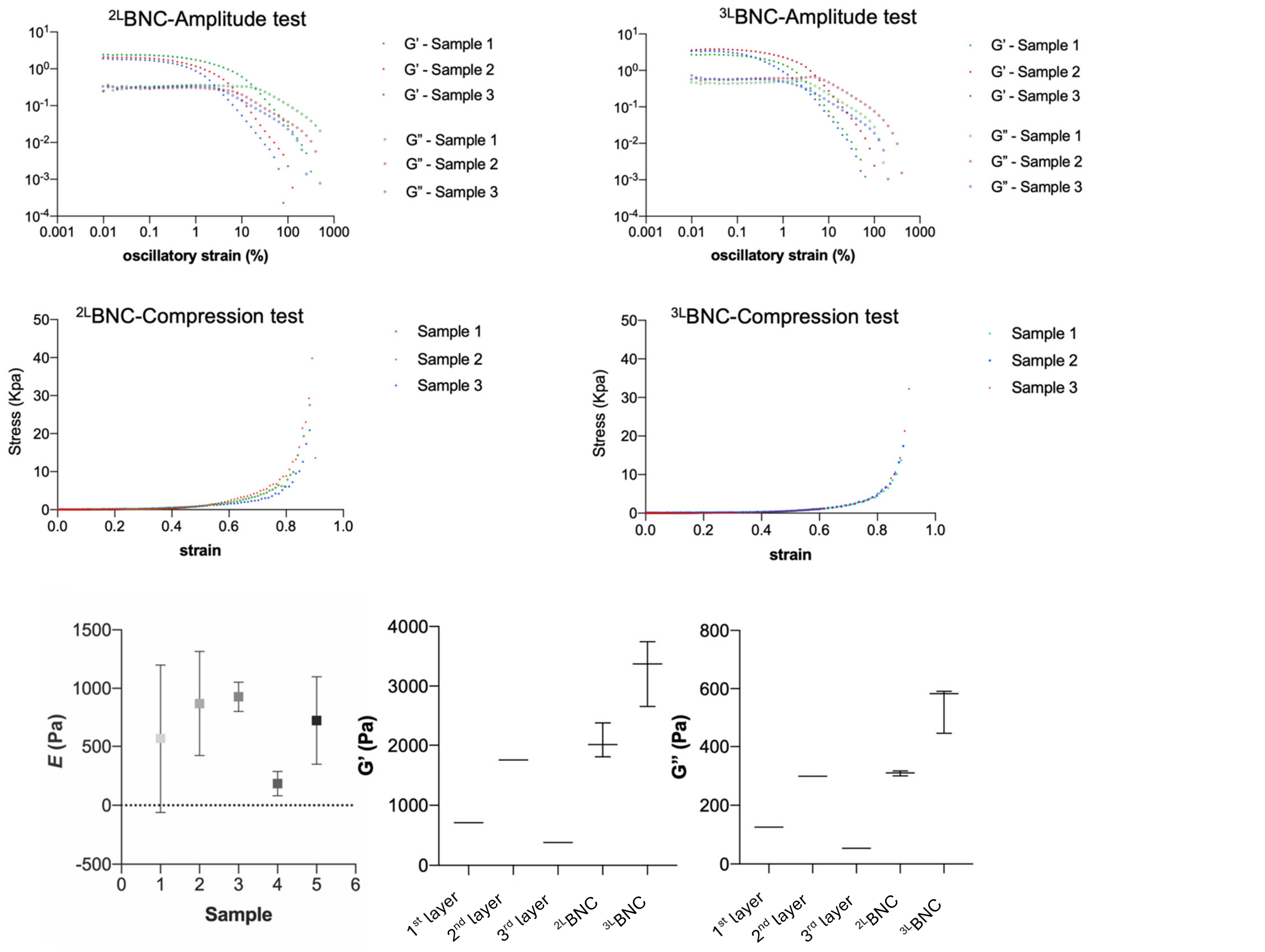

Figure S2: Graphs presenting rheology properties of the BNC scaffolds form which the average Young's modulus -E, Storage modulus – G'. Loss modulus-G" are calculated. n=3 for each sample.

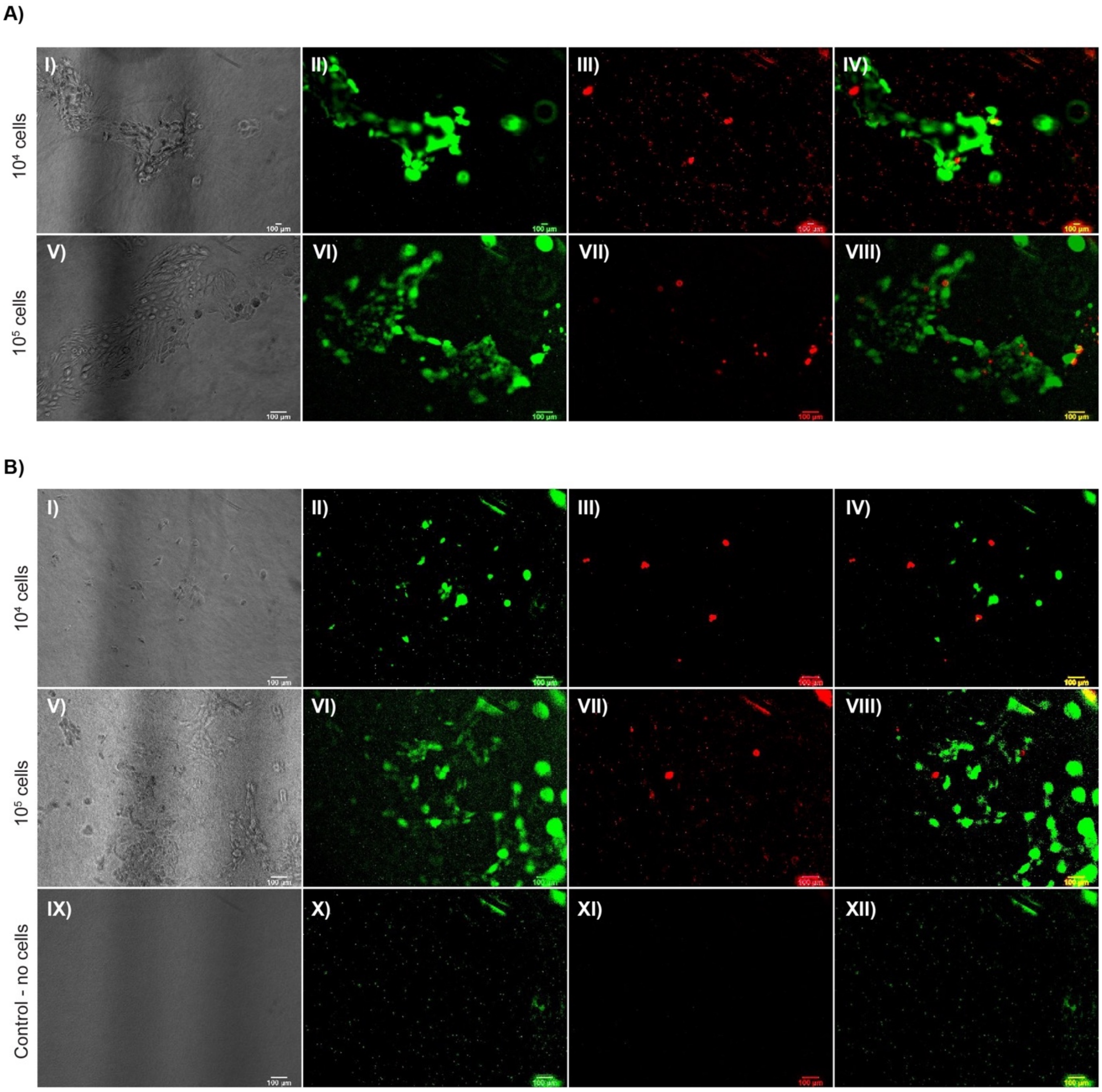

Figure S3: Live/Dead assay of one-week EOMA cells culture into the ^{2L}BNC (A) and into ^{3L}BNC (B) scaffolds at two different densities ($1x10^4$ and $1x10^5$ cells). Green: live cells; red: dead cells; last column: merged images. Scale bars: 100µm.

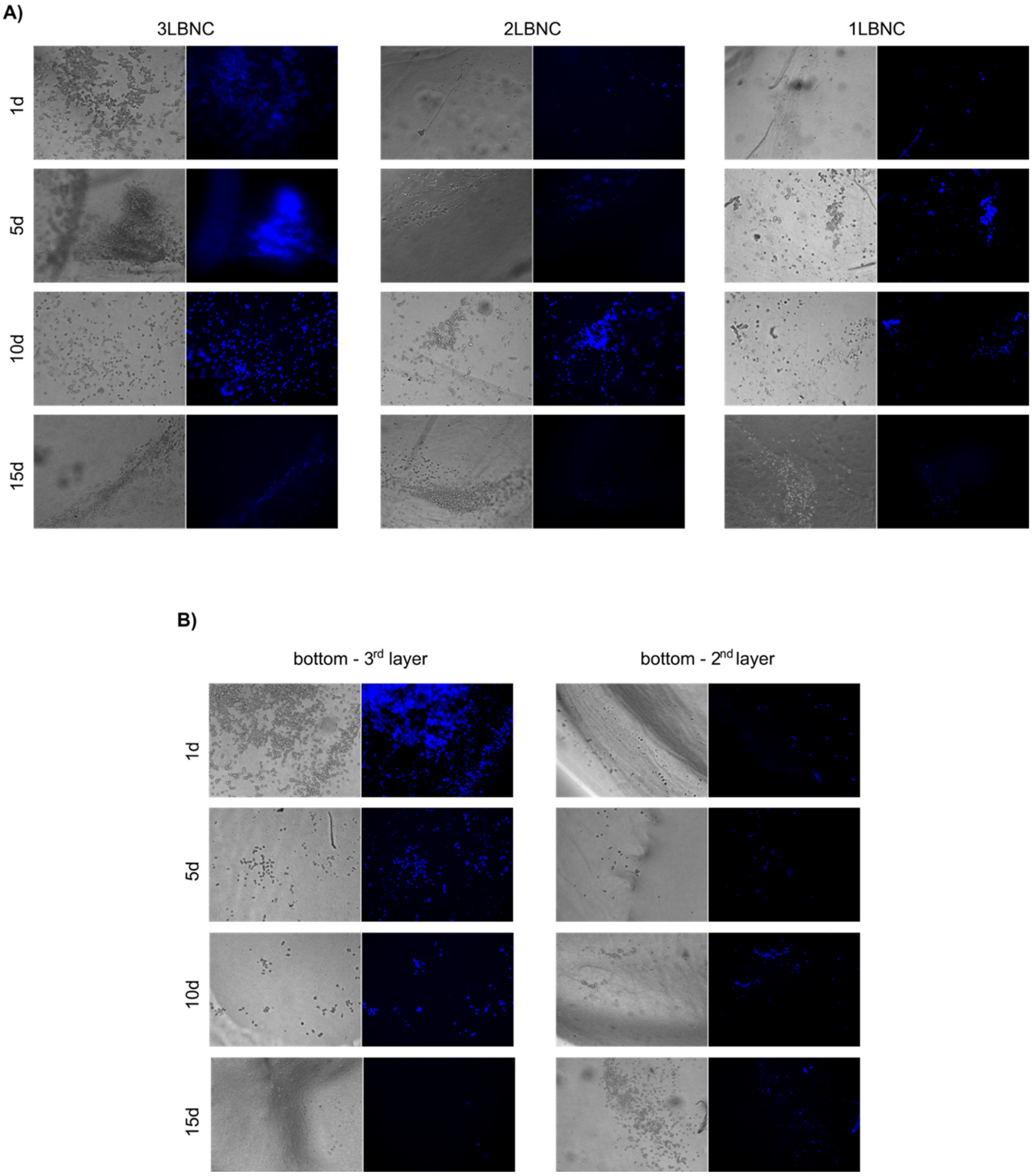

Figure S4: EA.hy926 cultured in the 3LBNC Scaffolds. 10^6 cells were injected in the second interlayer of the scaffold and cultured for 15 days. ^{3L}BNC samples remained inside inserts to avoid lateral cell migration. A) Images of the ^{3L}BNC, ^{2L}BNC and ^{SL}BNC, scaffolds, respectively. B) Images of underside of the third and the second individual layer removed from ^{3L}BNC.

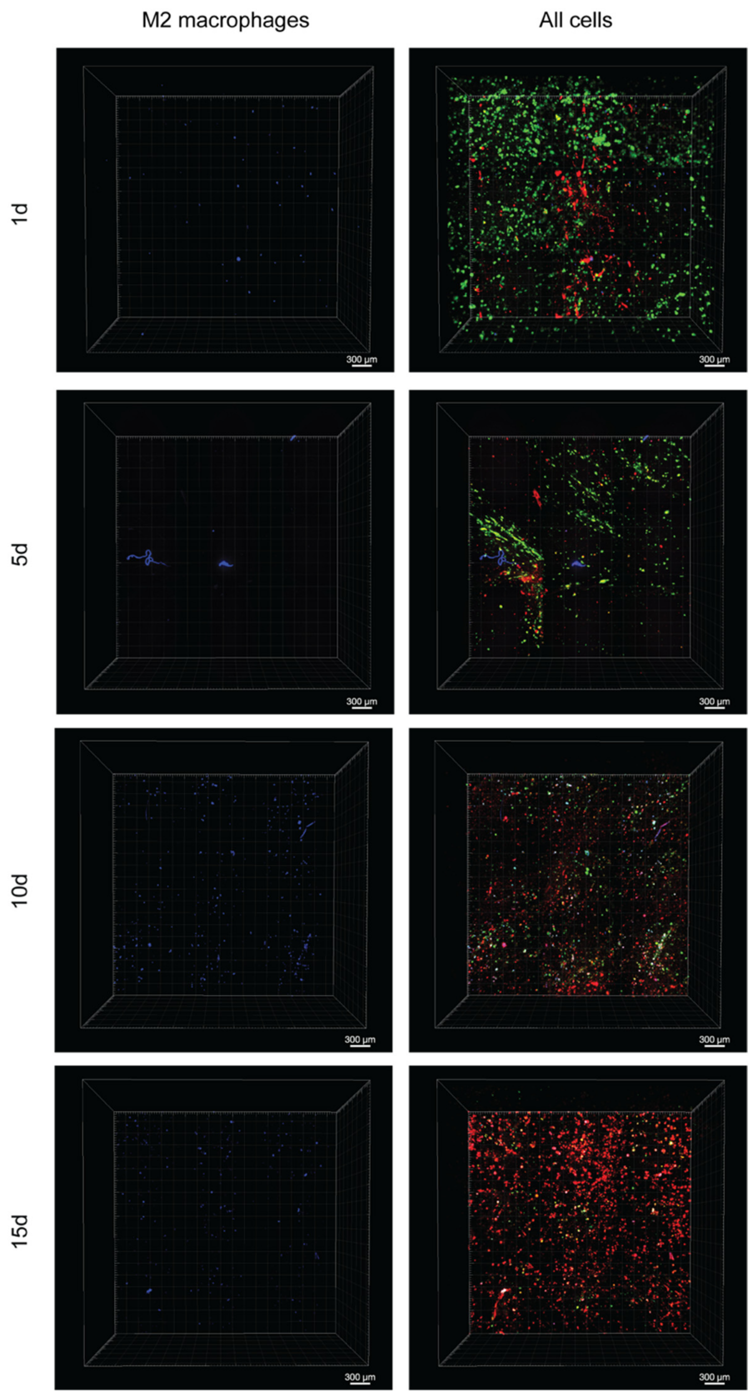

Figure S5: Images of M2 macrophages alone (first column) in comparison with their presence with all other cells simultaneously (second column) at different time-points: MDA-MB-231 in green, BC-CAFs in red and M2 macrophages in blue. Magnification 20x.

Table S2. Nanodrop results from RNA extraction by Trizol of cells grown in ^{3L}BNC Scaffold and cell culture plates. NC: negative control – no cells.

Culture in	Time-point	Concentration ng/µL	260/280	260/230
^{3L}BNC Scaffold	NC	5.9	1.67	0.10
	1d	7.9	1.54	0.08
	1d	63.1	1.79	0.29
	1d	80.1	1.87	0.33
	5d	70.0	1.87	0.47
	5d	222.4	1.86	0.73
	5d	117.1	1.86	0.59
	10d	219.6	1.85	1.22
	10d	167.6	1.91	0.79
	10d	22.4	1.94	0.21
	15d	172.5	1.91	0.87
	15d	235.0	1.91	1.10
	15d	38.6	1.83	0.27
Cell culture plates	NC	9.1	1.89	0.03
	1d	94.3	1.80	0.24
	5d	243.8	1.77	0.74
	5d	358.8	1.89	1.02
	10d	626.4	1.83	1.30
	10d	640.2	1.86	1.27
	15d	691.7	1.81	0.98
	15d	754.0	1.79	1.29
